# TGF-β1 Reduces Neutrophil Adhesion and Prevents Acute Vaso-Occlusive Processes in Sickle Cell Disease Mice

**DOI:** 10.3390/cells11071200

**Published:** 2022-04-02

**Authors:** Lidiane S. Torres, Hanan Chweih, Fernanda C. Z. Fabris, Erica M. F. Gotardo, Flávia C. Leonardo, Sara T. Olalla Saad, Fernando F. Costa, Nicola Conran

**Affiliations:** 1 Hematology and Transfusion Center, University of Campinas—UNICAMP, Campinas, São Paulo 13083-878, Brazil; 2 Ruth L. and David S. Gottesman Institute for Stem Cell and Regenerative Medicine Research, Albert Einstein College of Medicine, Bronx, New York, NY 10461, USA; 3 Department of Cell Biology, Albert Einstein College of Medicine, Bronx, New York, NY 10461, USA

**Keywords:** inflammation, vaso-occlusion, sickle cell anemia, neutrophils, vascular adhesion

## Abstract

Sickle cell disease (SCD) patients experience chronic inflammation and recurrent vaso-occlusive episodes during their entire lifetime. Inflammation in SCD occurs with the overexpression of several inflammatory mediators, including transforming growth factor beta-1 (TGF-β1), a major immune regulator. In this study, we aimed to investigate the role played by TGF-β1 in vascular inflammation and vaso-occlusion in an animal model of SCD. Using intravital microscopy, we found that a daily dose of recombinant TGF-β1 administration for three consecutive days significantly reduced TNFα-induced leukocyte rolling, adhesion, and extravasation in the microcirculation of SCD mice. In contrast, immunological neutralization of TGF-β, in the absence of inflammatory stimulus, considerably increased these parameters. Our results indicate, for the first time, that TGF-β1 may play a significant ameliorative role in vascular SCD pathophysiology, modulating inflammation and vaso-occlusion. The mechanisms by which TGF-β1 exerts its anti-inflammatory effects in SCD, however, remains unclear. Our in vitro adhesion assays with TNFα-stimulated human neutrophils suggest that TGF-β1 can reduce the adhesive properties of these cells; however, direct effects of TGF-β1 on the endothelium cannot be ruled out. Further investigation of the wide range of the complex biology of this cytokine in SCD pathophysiology and its potential therapeutical use is needed.

## 1. Introduction

Transforming growth factor-beta (TGF-β) is part of a highly conserved family of 33 members [1] that regulate numerous cell functions during development and adult tissue homeostasis [2]. TGF-β protein exists in three highly homologous isoforms—TGF-β1, TGF-β2, and TGF-β3—produced by virtually all circulating and tissue-resident leukocytes, and platelets [3,4]. All three isoforms participate in the start of development, but TGF-β1 is the master regulator of immunity after birth. TGF-β1 regulates recruitment, retention, apoptosis, differentiation, and proliferation of immune cells, enhancing or repressing inflammatory responses, depending on the cellular context [5,6]. Since the biological functions of TGF-β1 are highly context-dependent, the same cell type may respond to this cytokine in completely opposing manners under different situations [2]. In cancer, for example, TGF-β1 plays a dual role; while it inhibits cell proliferation and suppresses the early stages of tumor development, in later stages, it contributes to cancer progression and metastasis [7,8].

Increased circulating levels of TGF-β1 have been reported in sickle cell disease (SCD) [9,10], a severe genetic blood disorder characterized by chronic hemolytic anemia, vaso-occlusive episodes, progressive organ damage, and a reduced life expectancy [11,12]. Whether this cytokine is protective or detrimental to the pathophysiology of the disease, however, is unknown. The clinical complications of SCD arise from the polymerization of the mutated hemoglobin (Hb) S in the red blood cells (RBCs) [13,14]. Repeated RBCs sickling leads to the premature destruction of these cells in the circulation [15,16], promoting endothelial activation, with the expression of adhesion molecules, leukocyte recruitment and activation, and platelet activation and aggregation [17,18]. Cellular interactions involving sickle RBCs, leukocytes, platelets, and the endothelium can obstruct the blood flow, particularly in vessels of small caliber, promoting acute ischemia and restricting the blood supply to tissues [17,18,19].

Neutrophils are the most abundant leukocyte subset participating in SCD vaso-occlusion [20]. Activated neutrophils directly interact with endothelial E- and P-selectin, expressed by the activated endothelium, and have been shown to capture sickle RBCs via α_M_β_2_ integrin (Mac-1; CD11b) expression on the cell surface during TNFα-induced vascular inflammation in a mouse model of SCD [21,22]. Elevated leukocyte counts in the blood of SCD patients, mainly formed by neutrophils, have been associated with increased disease severity and risk of mortality [23,24]. As a key immune regulator that acts on a variety of cell types, TGF-β1 may conceivably play an important role in SCD by modulating the adhesive interactions of neutrophils with the endothelium. In this study, we investigated the effects of the neutralization and stimulation of TGF-β1 activity on microvascular inflammation in an animal model of SCD.

## 2. Materials and Methods

### 2.1. Reagents

Recombinant human TNFα, TGF-β1, and latent TGF-β1 protein were purchased from R&D Systems (Minneapolis, MN, USA). Anti-mouse TGF-β (clone 1D11.16.8) and anti-mouse IgG1 isotype control (clone MOPC-21) were purchased from Bio X Cell (West Lebanon, NH, USA). PercP-Cy5.5-conjugated mouse anti-human CD66b (clone G10F5), FITC-conjugated mouse anti-human CD11a (clone HI111), PE-conjugated mouse anti-human CD11b (clone ICRF44), PE-conjugated mouse anti-human CD62E (clone 68-5H11), and FITC-conjugated mouse anti-human CD106 (VCAM-1, clone 51-10C9) were purchased from BioLegend (San Diego, CA, USA). PE-conjugated anti-human CD11b (activation epitope-specific antibody, clone CBRM1/5), APC-conjugated CD62P monoclonal antibody (P-selectin, clone Psel.K02.3), and PERCP-conjugated ICAM-1 monoclonal antibody (clone 1H4) were purchased from eBioscience (Thermo Fisher Scientific, Waltham, MA, USA). All other reagents were purchased from Sigma-Aldrich (St. Louis, MO, USA) unless otherwise stated.

### 2.2. Animals

Male C57BL/6 mice were obtained from the animal breeding facility at the University of Campinas, Brazil, and were housed with a minimum of 2 and a maximum of 5 mice per cage, with free access to food and autoclaved water. Animals were fed on a 22% protein diet (NUVILAB—CR1 irradiated). Chimeric SCD mice and chimeric C57BL/6 mice were generated from the transplantation of nucleated bone marrow cells from Berkeley mice (SCD transgenic model; Tg(HuminiLCRα1GγAγδβS) Hba^−/−^Hbb^−/−^) or C57BL/6 mice into lethally irradiated C57BL/6 recipients (males, 8 weeks of age), as previously described [21,25]. Bone marrow reconstitution efficiency was analyzed three months after transplantation, by polyacrylamide gel electrophoresis [21]. Chimeric mice that originated from bone marrow transplantation are hereafter referred to as SCD mice and C57BL/6 mice. All experimental procedures were approved by the Animal Care and Use Committee of the University of Campinas (CEUA/UNICAMP; Protocol: 5067-1/2018) and performed in accordance with the Guide for the Care and Use of Laboratory Animals as adopted and promulgated by the US National Institutes of Health. All efforts were made to minimize animal suffering and to use the minimum number of animals necessary to produce replicable results.

### 2.3. In Vivo Treatments

SCD and C57BL/6 mice aged between 5–6 months were treated with monoclonal anti-TGF-β or recombinant human TGF-β1 protein. The latent recombinant human TGF-β1 was activated by acidification with 4 mM HCl solution containing 0.1% bovine serum albumin for 18 h prior to use, at 4 °C [26,27]. The mature human protein (active form) demonstrates 99% similarity to the murine active protein [1,28,29].

Mice received a daily dose of anti-TGF-β (100 µg in 200 µL PBS, i.p.) or active TGF-β1 (2 μg in 200 μL PBS, i.p.), on three consecutive days. As a control, a group of mice received saline (200 μL, i.p.), while another group received a non-specific isotype control antibody (IgG1 κ; 100 µg in 200 µL PBS, i.p.). Experiments were performed on the fourth day. Recombinant murine TNFα (0.5 µg in 200 µL PBS, i.p) was used as an inflammatory stimulus three hours before blood collection or intravital microscopy.

### 2.4. Intravital Microscopy

Mice were anesthetized with a mixture of xylazine (10 mg/kg) and ketamine (100 mg/kg), by intraperitoneal injection. The cremaster muscle was surgically exteriorized and visualized using an Axio Imager D2 microscope (Carl Zeiss Microscopy, Jena, Germany; 63X magnification) that was custom designed for intravital microscopy. The cremaster muscle was continuously superfused with bicarbonate-buffered saline (pH 7.4, 37 °C), and images of 6 to 10 venules (15–25 µm of diameter) per animal were recorded for 60 s (47 frames/s) using an Axiocam 503 monochromatic camera (Carl Zeiss Microscopy). The Rolling, adhesion, and extravasation of leukocytes were monitored for 30–45 min after surgery. Quantifications were made as previously described [25].

### 2.5. Human Samples

Peripheral blood samples from seven SCD patients (homozygous for HbS, i.e., HbSS, hereinafter referred to as SS) and six healthy individuals (homozygous for the normal adult hemoglobin A, i.e., HbAA, hereinafter referred to as AA), aged 28 to 53 years, were collected in heparin. Informed written consent was obtained from all individuals. This study was approved by the Ethics Committee of the University of Campinas, Brazil (Certificate of Presentation for Ethical Consideration: 78149417.5. *P* < 0.05 0.5404) and conducted in accordance with national guidelines for human research. None of the subjects had taken anti-inflammatory drugs during the two weeks preceding the sample collection. Six patients were being treated with hydroxyurea (HU; 15–30 mg/kg/day), and one patient had hydroxyurea use suspended six months before the sample collection date. Hematological parameters of SS patients are specified in Table S1.

### 2.6. Human Neutrophil Isolation

Human circulating neutrophils were isolated from the peripheral blood (~10 mL collected in heparin tubes) by density gradient centrifugation over Ficoll–Paque (Histopaque^®^) of densities of 1.077 g/mL and 1.119 g/mL [30]. The granulocyte layer was collected, and, after lysis of contaminating erythrocytes (155 mM NH_4_Cl, 10 mM KHCO_3_), cells were washed in PBS. Neutrophils were purified by CD66b-positive selection using magnetic beads (CD66abce MicroBead Kit, #130-092-393, Miltenyi Biotec, Auburn, CA, USA). Purity (>98%) was confirmed by flow cytometry (Figure S1A). Neutrophils (2 × 10^6^ cells/mL) were incubated with recombinant TGF-β1, or not, at concentrations of 1 ng/mL, 10 ng/mL, and 20 ng/mL for 2 h (37 °C, 5% CO_2_). For recombinant TNFα treatments, 100 ng/mL was added during the last 30 min of incubation.

### 2.7. Static Adhesion Assay

Static adhesion assays were performed as previously described [31]. Briefly, isolated neutrophils (2 × 10^6^ cells/mL in RPMI 1640 medium) were incubated in 96-well plates that were precoated with fibronectin ligand (20 µg/mL). Cells were allowed to adhere for 30 min at 37 °C, 5% CO_2_. Following incubation, non-adhered cells were discarded, and RPMI 1640 medium was added to the wells containing adhered neutrophils. A standard curve was built from the original cell suspension, and the percentage of cell adhesion was calculated by measuring the myeloperoxidase content of each well and comparing it to the standard curve.

### 2.8. Endothelial Cell Culture

Immortalized human umbilical vein endothelial cells (HUVECs) were obtained from the American Type Culture Collection (ATCC), Manassas, VA, USA. HUVECs were plated onto 24-well plates, at the concentration of 5 × 10^4^ cells/well in 500 μL of F12k medium containing 2% fetal bovine serum (FBS), 15 μg/mL growth factor, 10U heparin, and antibiotics. After 24 h of incubation at 37 °C and 5% CO_2_, the culture medium was changed, and HUVECs were treated with TGF-β1 (1, 10, or 20 ng/mL) for the next 2 h. In the 20th hour, 10 ng/mL TNFα was added as an inflammatory stimulus. At the end of the process, cells were detached by adding trypsin, followed by rapid neutralization with FBS. The harvested cells were washed in PBS and resuspended in the cultured medium for flow cytometry analysis. We performed three independent experiments with cells between the 17th and 18th passages.

### 2.9. Flow Cytometry

Neutrophils (2 × 10^6^ cells/mL in RPMI 1640 medium) were incubated with conjugated monoclonal antibodies for 30 min at 4 °C, protected from light. After incubation, cells were washed in PBS and submitted to flow cytometry acquisition (BD FACSCaliburTM, BD Biosciences, Jan Jose, CA, USA). A total of 5000 CD66b+ neutrophils were acquired in the neutrophil gate, whereby any eosinophils were excluded using forward scatter (FSC)/side scatter (SSC) gating (Figure S1A). The expression of β-integrins was determined by observing the expressions of the CD11a and CD11b subunits of the LFA-1 and Mac-1 integrins, respectively. The activity of CD11b was analyzed using CRBM1/5, a monoclonal antibody that reacts with an activation-specific epitope of the Mac-1 molecule. HUVECs plated on 24-well plates at a concentration of 5 × 10^4^ cells/well were harvested, washed in PBS, and incubated with conjugated monoclonal antibodies for 30 min at room temperature. A total of 5000 events were acquired and the expressions of the activation markers VCAM-1, ICAM-1, CD62E, and CD62P were analyzed. Data were analyzed by using the FlowJo ^TM^ v10.6 Software (BD Life Sciences).

### 2.10. Statistical Analysis

Hematological data and intravital microscopy data were analyzed by Student’s *t*-test or Mann–Whitney test, depending on whether they were parametric or nonparametric, respectively. Data generated from the in vitro assays with human neutrophils were analyzed using One-way repeated measures ANOVA, followed by the Bonferroni post hoc test. *P* < 0.05 was considered significant.

## 3. Results

### 3.1. TGF-β1 Administration Prevents Vaso-Occlusive Episodes in SCD Mice

**Figure 1:**
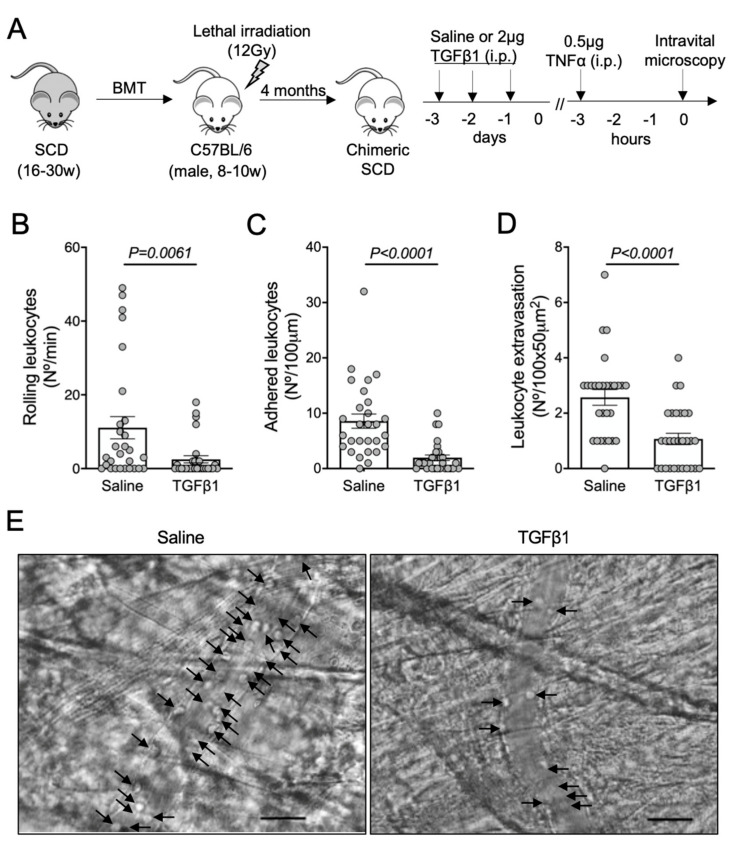
TNFα-induced vaso-occlusion in the microvasculature of SCD mice pre-treated with TGF-β1: (**A**) experimental design. Chimeric SCD mice were generated by transplantation of bone marrow cells (BMT) from transgenic SCD mice into lethally irradiated (12Gy) C57BL/6 male mice. After complete bone marrow repopulation (4 months), animals received TGF-β1 (2 μg, i.p) or saline as control for three consecutive days. On the fourth day, TNFα (0.5 μg, i.p.) was administered to induce acute vaso-occlusion. The microcirculation of the cremaster muscle was visualized by intravital microscopy; (**B**) number of rolling leukocytes (Nº/100 μm/min); (**C**) number of leukocytes adhered to endothelium (Nº/100 μm); (**D**) number of leukocytes extravasated to tissue (Nº/100 × 50 μm^2^). Saline group: *N =* 4 mice, 28 venules. TGF-β1 group: *N =* 3 mice, 29 venules. Mann–Whitney test for (**B**,**C**); Student’s *t*-test for (**D**); (**E**) representative images obtained during intravital microscopy (left: Video S1; right: Video S2). Arrows indicate leukocytes interacting with the endothelium (e.g., adhered or rolling). Scale bar = 20 μm.

To investigate a possible role for TGF-β1 in microvascular homeostasis in SCD, SCD mice were treated with 2 μg of recombinant TGF-β1 intraperitoneally for three consecutive days. On the fourth day, inflammation was induced with an intraperitoneal injection of TNFα, and intravital microscopy of the cremaster muscle was performed three hours later (Figure 1A). TGF-β1 significantly reduced the rolling (Figure 1B) and adhesion (Figure 1C) of leukocytes to the endothelium and prevented the extravasation of leukocytes through the adjacent tissue (Figure 1D). Representative images of intravital microscopy show a reduced number of leukocytes interacting with the endothelium in the microvasculature of a mouse treated with TGF-β1, compared with a mouse that received saline (Figure 1E; Videos S1 and S2). These results suggest that TGF-β1 is protective against TNFα-induced acute vaso-occlusion in SCD mice.

### 3.2. TGF-β1 Neutralization Exacerbates Vaso-Occlusion in SCD Mice

**Figure 2:**
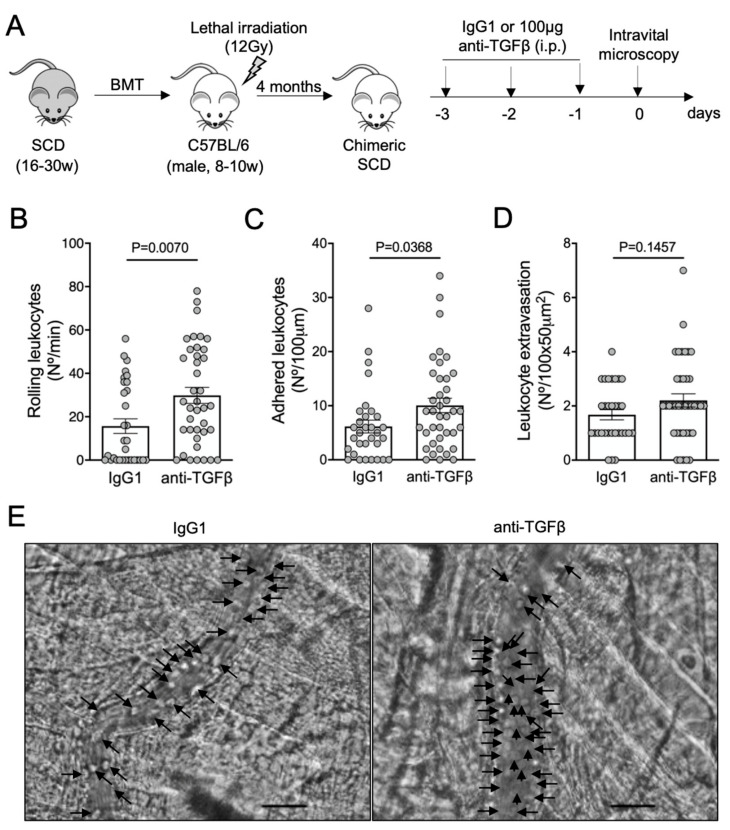
Vaso-occlusion in the microvasculature of SCD mice after neutralization of TGF-β: (**A**) experimental design. Chimeric SCD mice were generated by transplantation of bone marrow cells (BMT) from transgenic SCD mice into lethally irradiated (12Gy) C57BL/6 male mice. After complete bone marrow repopulation (4 months), animals were injected with anti-TGF-β antibody (100 μg, i.p) or IgG1 isotype control for three consecutive days. On the fourth day, the microcirculation of the cremaster muscle was visualized by intravital microscopy; (**B**) number of rolling leukocytes (Nº/100 μm/min); (**C**) number of leukocytes adhered to endothelium (Nº/100 μm); (**D**) number of leukocytes extravasated to tissue (Nº/100 × 50 μm^2^). IgG1 group: *N* = 4 mice, 31 venules. Anti-TGF-β group: *N* = 5 mice, 39 venules. Student’s *t*-test for (**B**,**C**); Mann–Whitney test for (**D**); (**E**) representative images obtained during intravital microscopy (left: Video S3; right: Video S4). Arrows indicate leukocytes interacting with the endothelium (e.g., adhered or rolling). Scale bar = 20 μm.

To confirm the protective role of TGF-β1 in microvascular inflammation, we treated SCD mice with a monoclonal antibody able to neutralize the TGF-β isoforms 1, 2, and 3. Mice received a daily intraperitoneal dose of anti-TGF-β (100 μg) or an isotype control IgG1 (100 μg), for three consecutive days. On the fourth day, we analyzed the cremaster microcirculation by intravital microscopy. No further inflammatory or anti-inflammatory stimuli were used (Figure 2A). Neutralization of the TGF-β isoforms, without any additional inflammatory challenge, significantly exacerbated the rolling (Figure 2B) and the adhesion (Figure 2C) of leukocytes in the microvasculature of SCD mice, compared with mice treated with an IgG1 control antibody. Leukocyte extravasation, however, did not statistically differ between mice treated with IgG1 (1.7 ± 0.2 cells/field) and anti-TGF-β (2.2 ± 0.2 cells/field) (*P* = 0.15, Figure 2D). Representative images of intravital microscopy show a higher number of leukocytes interacting with a venule of a mouse treated with anti-TGF-β, compared with a mouse treated with the isotype control IgG1 (Figure 2E; Videos S3 and S4). These results suggest that TGF-β is required for microvascular homeostasis in SCD mice. As TGF-β1 is the most abundant of the three TGF-β isoforms in mammals [1], we attributed the worsening of vaso-occlusion parameters in our SCD mouse model, following anti-TGF-β administration, mainly to the absence of functional TGF-β1.

### 3.3. TGF-β1 Is Required for Microvascular Homeostasis in Healthy C57BL/6 Mice

**Figure 3:**
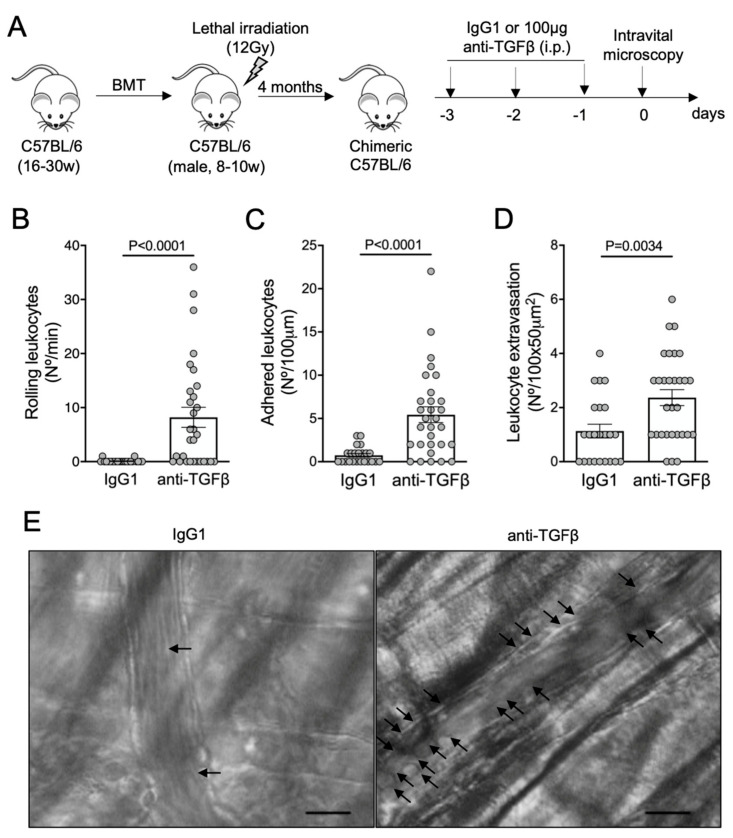
Vaso-occlusion in the microvasculature of C57BL/6 mice after neutralization of TGF-β: (**A**) experimental design. Chimeric C57BL/6 mice were generated by transplantation of bone marrow cells (BMT) from C57BL/6 donors into lethally irradiated (12Gy) C57BL/6 male recipients. After complete bone marrow repopulation (4 months), animals were injected with anti-TGF-β antibody (100 μg, i.p.) or IgG1 isotype control for three consecutive days. On the fourth day, the microcirculation of the cremaster muscle was visualized by intravital microscopy; (**B**) number of rolling leukocytes (Nº/100 μm/min); (**C**) number of leukocytes adhered to endothelium (Nº/100 μm); (**D**) number of leukocytes extravasated to tissue (Nº/100 × 50 μm^2^). IgG1 group: *N* = 4 mice, 23 venules. Anti-TGF-β group: *N* = 4 mice, 30 venules. Mann–Whitney test for (**B**,**C**); Student’s *t*-test for (**D**); (**E**) representative images obtained during intravital microscopy (left: Video S5; right: Video S6). Arrows indicate leukocytes interacting with the endothelium (e.g., adhered or rolling). Scale bar = 20 μm.

To address whether the neutralization of TGF-β could promote vaso-occlusion under non-inflammatory conditions, we subjected C57BL/6 wild-type mice to the same anti-TGF-β protocol (Figure 3A). To avoid any bias due to the bone marrow transplantation procedure, our C57BL/6 mice were also lethally irradiated and transplanted with bone marrow cells from C57BL/6 donors. The rolling (Figure 3B), adhesion (Figure 3C), and extravasation (Figure 3D) of leukocytes were drastically augmented by the blocking of TGF-β1 in C57BL/6 mice. Images of intravital microscopy reveal numerous adherent leukocytes in a venule from an anti-TGF-β-treated mouse, in contrast to the non-inflamed venule from an IgG1-treated mouse (Figure 3E; Videos S5 and S6). TNFα-induced vaso-occlusion was also prevented in the venules of C57BL/6 mice pretreated with recombinant TGF-β1 (Figure S1A–E, Videos S7 and S8).

### 3.4. TGF-β1 Reduces TNFα-Induced Adhesion of Human Neutrophils to Fibronectin In Vitro

**Figure 4:**
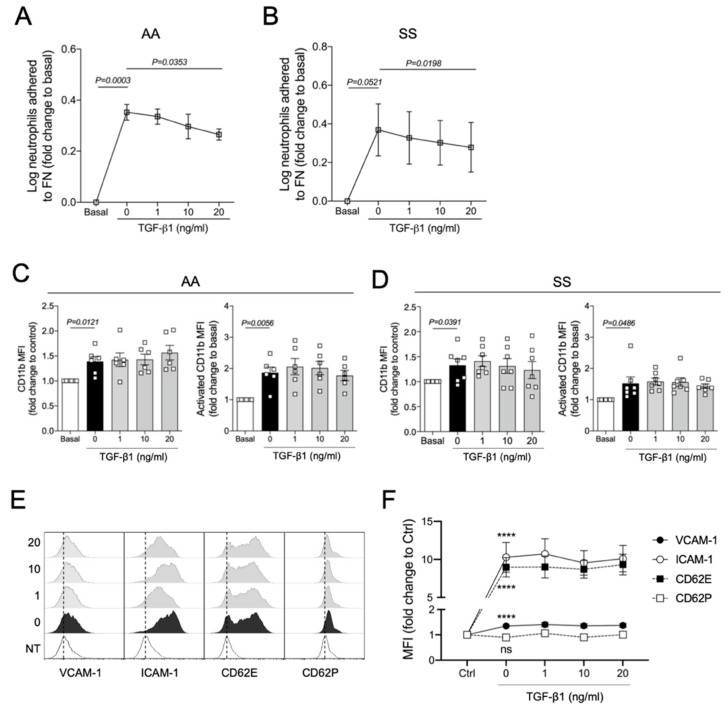
In vitro effects of TGF-β1 on human neutrophils and immortalized endothelial cells: (**A**) TNFα-induced adhesion of control human neutrophils (AA, *N* = 5) and (**B**) neutrophils from SCD patients (SS, *N* = 5) to fibronectin (FN) under different TGF-β1 treatments; (**C**) TNFα-induced expression and activation of CD11b on neutrophils from controls (AA, *N* = 6) and (**D**) SCD individuals after treatment with different concentrations of TGF-β1 (SS, *N* = 7)’ (**E**) Histograms illustrating the TNFα-induced expression of endothelial activation markers by HUVECs incubated with different concentrations of TGF-β1 (dashed lines are used as a reference for the MFI displayed by untreated HUVECs); (**F**) expression of endothelial molecules expressed on TNFα-activated HUVECs under different TGF-β1 concentrations. Neutrophils (2 × 10^6^/mL in 500 μL RPMI medium) were treated with TGF-β1 at three different concentrations: 1 ng/mL, 10 ng/mL, or 20 ng/mL for 90 min, and stimulated with 200 ng/mL TNFα for 15–30 additional minutes. For (**A**,**B**), cells were allowed to adhere to an FN (20 µg/mL) precoated plate for 30 min (37 °C, 5% CO_2_). The percentage of adhered cells was calculated. For (**C**,**D**), neutrophils were stained with conjugated anti-human PercP-CD66b, PE-CD11b; or with PercP-CD66b and PE-CD11b (activated epitope). For (**E**,**F**), HUVECs (5 × 10^4^ cells in F12K medium) were added into 24-well plates and allowed to adhere for 24 h (37 °C, 5% CO_2_). Cells were then treated with three concentrations of recombinant human TGF-β1 (1, 10, and 20 ng/mL). After 20 h they received an inflammatory stimulus with 10 ng/mL TNFα for 4 h. At the end of the 48 h of incubation, cells were harvested (trypsinization) and stained with anti-human conjugated FITC-CD106 (VCAM-1), PercP-ICAM-1, APC-CD62P, and PE-CD62E. Statistical comparisons were performed against untreated cells (basal or NT, no treatment) and TNFα-activated cells with no TGF-β1 (0 ng/mL). One-way repeated-measures ANOVA was used, followed by Bonferroni post hoc test. ****, *P* < 0.0001.

Although our findings demonstrate the protective effects of TGF-β1 on acute vaso-occlusive processes in SCD mice, whether this cytokine acts on the endothelium or leukocytes is not clear. It has been demonstrated that 90% of the leukocytes interacting with the endothelium in the microvasculature of SCD mice, visualized in intravital microscopy, are neutrophils [20]. To address whether TGF-β directly affects the adhesive properties of these cells, we conducted static cell adhesion assays using human neutrophils. We investigated the adhesive capacity of TNFα-stimulated neutrophils, preincubated (or not) with three concentrations of recombinant TGF-β1 (1, 10, or 20 ng/mL). As a ligand, we used fibronectin, an extracellular matrix protein for which β-integrins have a strong binding affinity. We observed that the adhesions of neutrophils from both control individuals (AA, Figure 4A) and SS patients (Figure 4B) to FN were increased by TNFα, and this adhesion was slightly but significantly abrogated by TGF-β1 at a concentration of 20 ng/mL. These results suggest that the improvement in the vaso-occlusive parameters observed in vivo could be partially explained by the direct action of TGF-β1 on the adhesiveness of neutrophils.

Next, the expression of α2-integrins LFA-1 (α_L_β_2_; CD11a/CD18) and Mac-1 (α_M_β_2_; CD11b/CD18) on neutrophils was assessed by flow cytometry using anti-human CD11a and CD11b antibodies. As expected, TNFα not only induced the expression of Mac-1 on AA and SS neutrophils but also increased its activation (Figure 4C,D). However, no effect of TGF-β1 on TNFα-induced Mac-1 expression and activity was observed in our in vitro model (Figure 4C,D). LFA-1 expression was not significantly stimulated or inhibited by any of our treatment conditions (Figure S2B).

To access the role of TGF-β1 in the endothelium, immortalized human umbilical vein endothelial cells (HUVECs) were pretreated with TGF-β1 and challenged with TNFα. The expressions of vascular cell adhesion protein 1 (VCAM-1), intracellular adhesion molecule 1 (ICAM-1), P-selectin (CD62P), and E-selectin (CD62E) were evaluated by flow cytometry (Figure 4E,F). TNFα significantly induced the expression of VCAM-1, ICAM-1, and CD62E by HUVECs; however, preincubation with TGF-β1 did not prevent the activation of endothelial adhesion molecules induced by TNFα.

## 4. Discussion

TGF-β family-related proteins play multiple roles in the development, tissue differentiation, and maintenance of homeostasis [3,32]. Herein, we showed that administration of TGF-β1 reduced the acute microvascular inflammation triggered by TNFα in SCD mice, while the neutralization of TGF-β drastically increased the adhesion, rolling, and extravasation of leukocytes. Previous studies have shown that depletion or disturbance of TGF-β1 signaling results in autoimmune disorders or even lethal inflammation in mice [33,34], while the stimulation of TGF-β1 promotes anti-inflammatory effects in bowel disease [35].

The microcirculation of a noninflamed model (C57BL/6, wild-type mice) was compromised when TGF-β was neutralized, with a significant promotion of adhesion, rolling, and transmigration of leukocytes. The interactions observed between leukocytes and endothelial cells in healthy mice after treatment with anti-TGF-β indicate that this cytokine is required for the maintenance of vascular homeostasis in both healthy and pathological conditions.

Neutrophils are a crucial component of SCD pathophysiology, and their interactions with activated platelets, sickle RBCs, and endothelium can trigger vaso-occlusive events [36,37,38]. Our in vitro studies evidenced a slight but significant direct effect of TGF-β1 on human neutrophil adhesion, which was not related to the expression of beta-integrins. Other in vitro studies have shown that TGF-β1 could prevent neutrophil degranulation and activation [39], as well as neutrophil adhesion [40] and migration [41], through the activated endothelium.

It has been demonstrated that TGF-β1 can also inhibit the adhesion of neutrophils on endothelial cells [40] by directly acting on the endothelium, reducing the expression of E-selectin, with no change in VCAM-1 or ICAM-1 expression [42]. In our study, however, TGF-β1 failed to prevent the TNFα-induced activation of immortalized HUVECs. According to Gamble et al., (1988) [42], the responsiveness of endothelial cells to TGF-β decreases over time of incubation after explantation from umbilical veins, which may justify our results with HUVECs at passages 17 to 18. Therefore, the hypothesis that TGF-β1 signaling may impact both leukocytes and endothelial cells during vaso-occlusion cannot be ruled out.

One of the most notable features of TGF-β1 is its pleiotropic nature, which is highly dependent on the context and crosstalk with other signaling pathways. The biological effects of TGF-β are contextual, and even the same cell type may show different or opposing responses to the ligand under distinct biological contexts. Co-stimulation by other cytokines also modifies the responsiveness to TGF-β1 [2]. Perturbations in TGF-β signaling or loss of TGF-β signaling components cause vascular pathologies, including aneurysms, atherosclerosis, retinopathy, endothelial tumors, and cardiovascular disease [43,44]. Mutations affecting the TGF-β pathway have also been associated with gastrointestinal cancers resulting from chronic and exacerbated inflammation [45]. Specifically, in SCD, a severe inflammatory condition associated with endothelial dysfunction, we demonstrate that TGF-β1 promotes vascular and immune homeostasis by reducing the adhesion of neutrophils to the endothelium and the severity of the vaso-occlusive processes. Whether TGF-β1 is acting alone or in synergy with other inflammatory mediators, however, needs further investigation.

## Data Availability

Not applicable.

## Supplementary Materials

The following supporting information can be downloaded at: https://www.mdpi.com/article/10.3390/cells11071200/s1, Table S1: Hematological parameters of the SCD patients (SS), Figure S1: TNFα-induced vaso-occlusion in the microvasculature of C57BL/6 mice pretreated with TGF-β1, Figure S2: In vitro effects of TGF-β1 on CD11a expression by human neutrophils, Video S1: Representative intravital microscopy video of a venule from SCD mouse treated with saline and challenged with TNFα, Video S2: Representative intravital microscopy video of a venule from SCD mouse treated with recombinant TGF-β1 and challenged with TNFα, Video S3: Representative intravital microscopy video of a venule from SCD mouse treated with isotype IgG1, Video S4: Representative intravital microscopy video of a venule from SCD mouse treated with anti-TGF-β, Video S5: Representative intravital microscopy video of a venule from a C57BL/6 mouse treated with isotype IgG1, Video S6: Representative intravital microscopy video of a venule from a C57BL/6 mouse treated with anti-TGF-β, Video S7: Representative intravital microscopy video of a venule from a C57BL/6 mouse treated with saline and challenged with TNFα, Video S8: Representative intravital microscopy video of a venule from a C57BL/6 mouse treated with recombinant TGF-β1 and challenged with TNFα.
