# Supplementary material for: TGF-β1 Reduces Neutrophil Adhesion and Prevents Acute Vaso-Occlusive Processes in Sickle Cell Disease Mice
Source: Cells. 2022 Apr 2;11(7):1200. doi: 10.3390/cells11071200 (PMC8998040; doi:10.3390/cells11071200)
Supplement: Supplementary file 1 [file cells-11-01200-s001.zip › cells-1651078-supplementary.pdf]

Table S1. Hematological parameters of the SCD patients (SS).

Parameters	Presentation	Values
Age (years)	Median, min-max	44, 35-53
Gender (nº)	Male / Female	4 / 3
HU use (nº)	Off	7 *
RBC (10^6/µL)	Mean ± SD	2.47 ± 0.54
Hemoglobin (g/dL)	Mean ± SD	9.41 ± 1.64
HbF (%)	Mean ± SD	14.76 ± 6.82
WBC (10^3/µL)	Mean ± SD	6.23 ± 1.83
Neutrophils (10^3/µL)	Mean ± SD	3.14 ± 1.05
Platelets (10^6/µL)	Mean ± SD	392.86 ± 66.70

HU, hydroxyurea; RBC, red blood cell; HbF, fetal hemoglobin; WBC, white blood cell; min, minimum; max, maximum; SD, standard deviation. * one patient had HU use suspended six months before sample collection.

Figure S1:

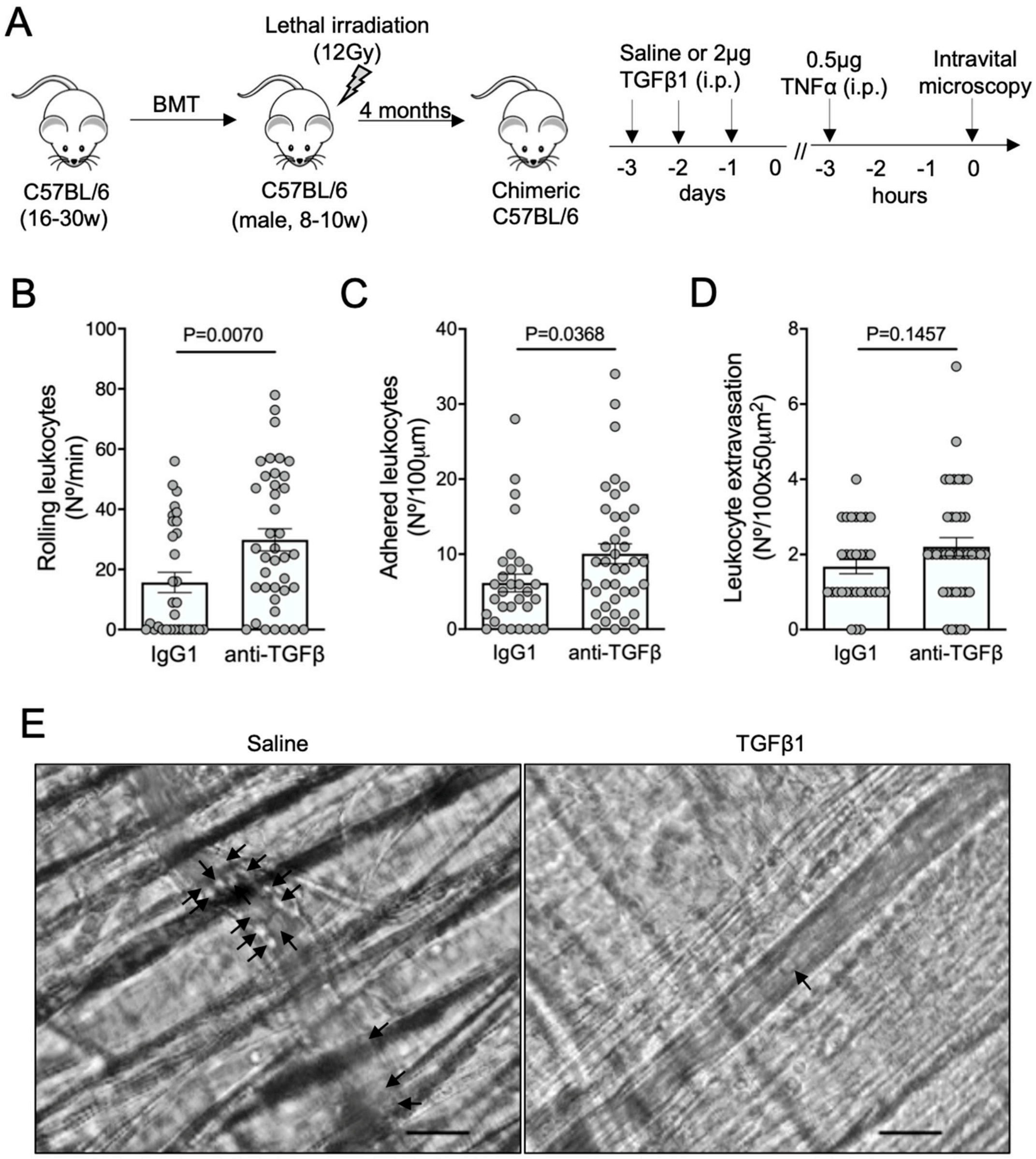

Figure S1. TNFα-induced vaso-occlusion in the microvasculature of C57BL/6 mice pretreated with TGF-β1: (A) experimental design. Chimeric C57BL/6 mice were generated by transplantation of bone marrow cells (BMT) from C57BL/6 donors into lethally irradiated (12Gy) C57BL/6 male recipients. After complete bone marrow repopulation (4 months), animals received TGF-β1 (2 µg, i.p.) or saline as control

for three consecutive days. On the fourth day, TNFα (0.5 μg, i.p.) was administered to induce acute vaso-occlusion. The microcirculation of the cremaster muscle was visualized by intravital microscopy; **(B)** number of rolling leukocytes (Nº/100 μm/min); **(C)** number of leukocytes adhered to endothelium (Nº/100 μm)**; (D)** number of leukocytes extravasated to tissue (Nº/100 × 50μm^2). Saline group: N=3 mice, 25 venules. TGFβ1 group: N=3 mice, 26 venules. Mann–Whitney test for B; Student's *t*-test for C and D; **(E)** representative images obtained during intravital microscopy (left: Video S7; right: Video S8). Arrows indicate leukocytes interacting with the endothelium (e.g., adhered or rolling). Scale bar = 20 μm.

Figure S2:

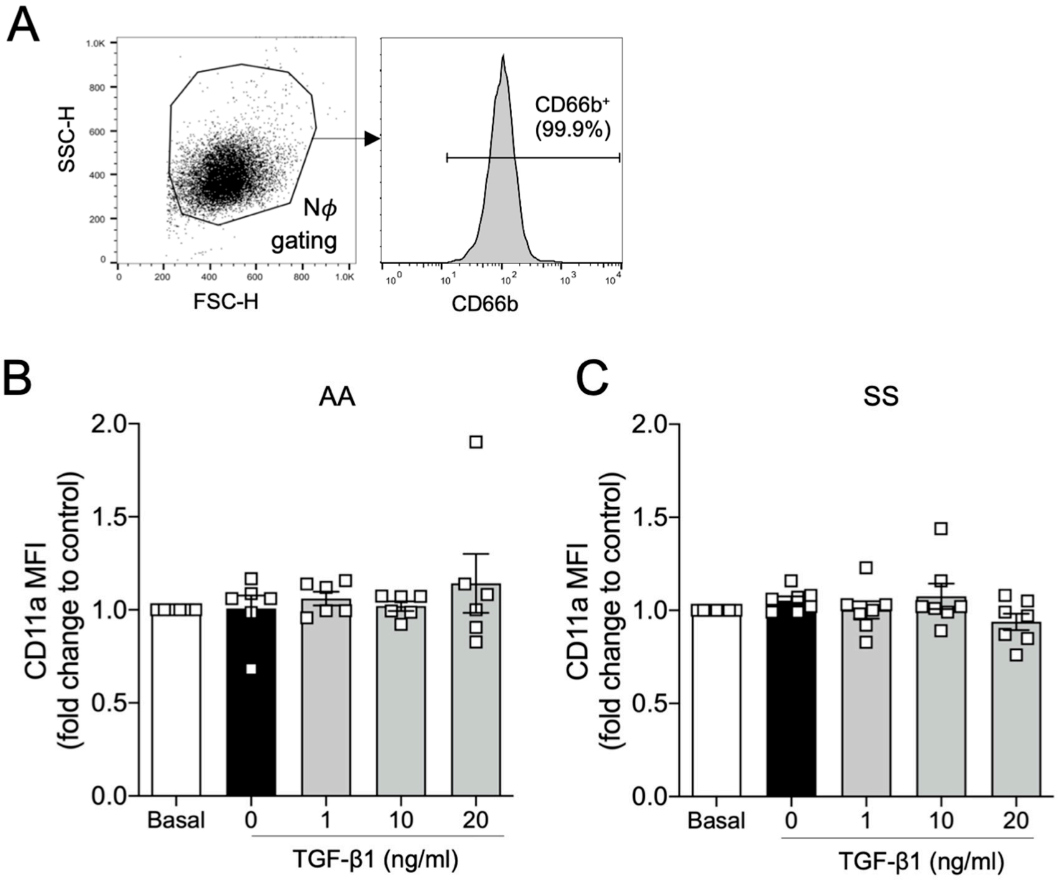

Figure S2. In vitro effects of TGF-β1 on CD11a expression by human neutrophils: (A) flow cytometry dot plot and histogram demonstrating the purity of neutrophils (NØ; >99%); **(B)** TNFα-induced expression of CD11a (e.g., LFA-1 integrin) on neutrophils from controls (AA, N=6) and **(C)** SCD patients (SS, N=7) after treatment with TGF-β1. Neutrophils (2×10^6/mL in 500 μL of RPMI 1640 medium) were treated with TGF-β1 at three concentrations: 1 ng/mL, 10 ng/mL, or 20 ng/mL for 90 min, and stimulated with 200 ng/mL TNFα for 30 additional minutes. Cells were stained with conjugated anti-human PercP-CD66b and FITC-CD11a. Statistical comparisons were performed against untreated cells (basal) and TNFα-activated cells with no TGF-β1 (0 ng/mL). One-way repeated-measures ANOVA.

Video Legends

Video S1: Representative intravital microscopy video of a venule from SCD mouse treated with saline and challenged with TNFα. Scale bar = 20 μm.

Video S2: Representative intravital microscopy video of a venule from SCD mouse treated with recombinant TGF-β1 and challenged with TNFα. Scale bar = 20 μm.

Video S3: Representative intravital microscopy video of a venule from SCD mouse treated with isotype IgG1. Scale bar = 20 μm.

Video S4: Representative intravital microscopy video of a venule from SCD mouse treated with anti-TGF-β. Scale bar = 20 μm.

Video S5: Representative intravital microscopy video of a venule from a C57BL/6 mouse treated with isotype IgG1. Scale bar = 20 μm.

Video S6: Representative intravital microscopy video of a venule from a C57BL/6 mouse treated with anti-TGF-β. Scale bar = 20 μm.

Video S7: Representative intravital microscopy video of a venule from a C57BL/6 mouse treated with saline and challenged with TNFα. Scale bar = 20 μm.

Video S8: Representative intravital microscopy video of a venule from a C57BL/6 mouse treated with recombinant TGF-β1 and challenged with TNFα. Scale bar = 20 μm.
